# Insect cell culture in reagent bottles

**DOI:** 10.1016/j.mex.2014.08.006

**Published:** 2014-08-28

**Authors:** S. Rieffel, S. Roest, J. Klopp, S. Carnal, S. Marti, B. Gerhartz, B. Shrestha

**Affiliations:** Protein Science Group, Novartis Institute of Biomedical Research, Center for Proteomic Chemistry, Basel, Switzerland

**Keywords:** Insect cell, Baculovirus, BV, Sf9, Sf21, High-throughput, Fermentation vessel, Reagent bottle, High volume culture, Expression

## Abstract

Growing insect cells with high air space in culture vessel is common from the early development of suspension cell culture. We believed and followed it with the hope that it allows sufficient air for optimal cell growth. However, we missed to identify how much air exactly cells need for its growth and multiplication. Here we present the innovative method that changed the way we run insect cell culture. The method is easy to adapt, cost-effective and useful for both academic and industrial research labs. We believe this method will revolutionize the way we run insect cell culture by increasing throughput in a cost-effective way.

In our study we identified:

• Insect cells need to be in suspension; air space in culture vessel and type of culture vessel is of less importance. Shaking condition that introduces small air bubbles and maintains it in suspension for longer time provides better oxygen transfer in liquid. For this, high-fill volume in combination with speed and shaking diameter are important.
• Commercially available insect cells are not fragile as original isolates. These cells can easily withstand higher shaking speed.
• Growth condition in particular lab set-up needs to be optimized. The condition used in one lab may not be optimum for another lab due to different incubators from different vendors.

## Graphical abstract

**Figure:**
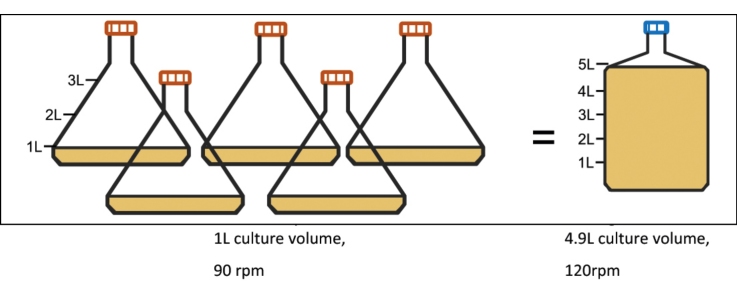

## Method details

Growth of insect cells in reagent bottle serves better alternative to carry out multi-parallel cultures in volume ranges from 0.25 L to 5 L.

## Preparation of reagent bottles for cell culture

1. Reagent bottles (Cat. no. 100389, VITLAB) with closed cap are delivered non-sterile from vendor. Cap was removed and the opening was covered with aluminum foil.
2. BOLA screw-caps (Article No. H 999-45, Bola) were placed either in sterilizing bag or suitable container.
3. Bottles and caps were sterilized by autoclaving (121 °C, 15 psi, 15 min).
4. After sterilization, aluminum foil was removed and replaced with BOLA-caps under laminar flow in a hood. The opening of caps was covered with AirPore tape sheet (Cat. no. 19571, Qiagen).
5. Alternatively, AirPore tape can be used directly on bottle. Using BOLA caps make it easy to take samples during culture if necessary.
6. Bottles can be sterilized with loosened BOLA caps on, covered with aluminum foil. After sterilization, aluminum foil can be replaced with AirPore tape.

## Routine growth and maintenance of cells

1. Sf9 or Sf21 cells adapted to suspension culture in Sf-900™ III SFM medium (Cat. no. 12658-027, life technologies) were grown to log phase, i.e. maintaining viable count in the range of (2–5) × 10^6^ cells per ml and viability greater than 95%. This serves as a master stock.
2. An aliquot of cells were taken from the master stock and diluted with fresh medium to initiate 50 ml starter culture in 250 ml Erlenmeyer flask (Cat. no. 431144, Corning^®^). Recommended starting density is (0.7–0.8) × 10^6^ cells per ml.
3. Diluted culture was incubated at 27 °C with 90 rpm in shaker incubator (Kuhner ISF-4-V: 50 mm rotating diameter).
4. Cell count and viability were analyzed using Vi-Cell™ XR cell counter (Beckman Coulter). It is recommended to check cell parameters every day for first few days which will give an idea on frequency of passage required. We passage cells two times a week, on day 3 and day 7.
5. Cells were passaged once the viable count reaches approximately (8–9) × 10^6^ cells per ml by adding fresh medium to density of (0.7–0.8) × 10^6^ cells per ml, as mentioned in point 2 above.

*Note*: If you are new to cell culture and starting your first cell culture in suspension, please follow supplier's instruction to revive cells from frozen stock.

## Growth of cells in different culture vessel

In order to establish correlation of growth among different culture vessel, four experimental setups were designed (see Table 1). The vessels used were:

• Tubespin^®^ Bioreactor 50 (Cat. no. 87050, TPP Techno Plastic Product),
• Erlenmeyer flask (Cat. no. 431144, Corning^®^), and
• Bottle (Cat. no. 100389, VITLAB).

*Note*: Tubespin^®^ Bioreactor 50 is used for the small scale expression study for culture volume from 5 ml to 30 ml.

Sf21 cells were used to establish the method since these cells are considered more fragile than Sf9.

1. Pre-culture of Sf21 in Sf900III medium (about 200 ml) was initiated with starting density of ∼0.8 × 10^6^ cell per ml. After 48 h, cells were in mid-log phase i.e. cell count was around 3 × 10^6^ cells per ml.
2. Culture was diluted to 0.8 × 10^6^ cells per ml by mixing 160 ml of pre-culture and 440 ml of fresh medium.
3. Diluted culture was distributed in different culture vessel and incubated at 27 °C as indicated in Table 1.

**Fig. 1:**
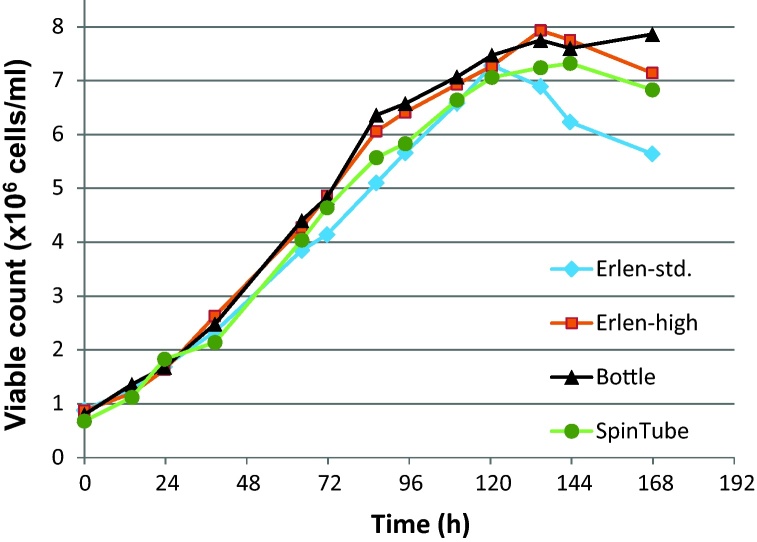
Monitoring growth and multiplication of Sf21 insect cell line in different culture vessels.

Growth of cells was measured over different time point for 168 h (7 days). A graph of viable cell count was plotted against time (Fig. 1). There was no significant difference in viable count in all cases until 72 h. It is worth mentioning that 48–72 h of growth is important in terms of cell maintenance and heterologous protein expression. Other cells parameters such as total count, viability and diameter were also observed at each time point and no significant differences were observed over the time.

This confirms the possible use of all 3 vessels for routine growth and maintenance of insect cells. It further confirms that high ratio of vessel volume (V1) versus culture volume (V2) is of less importance to keep optimum cell growth. Similar behavior is observed in case of Sf9 insect cells (data not shown). It is evident from Fig. 1 that small Erlenmeyer flasks can be used for high-fill culture, however, use of large Erlenmeyer flask such as 3 L flask with high-fill culture was not suitable due to wide base and tapered neck that limits aeration.

## Growth behavior over different passages

Bottles and Tubespins were chosen to observe growth behavior over several passages.

1. Pre-culture of Sf9 and Sf21 in Sf900III medium was prepared as mentioned in previous section.
2. Culture was diluted to 0.8 × 10^6^ cells/ml. 300 ml of dilute culture was prepared to dispense 250 ml in a bottle and 30 ml in a TubeSpin.
3. It was incubated at 27 °C with 230 rpm.
4. Cell parameters (total count, viable count, viability and cell diameter) were measured using Vi-Cell at two different time point in a day.
5. Once cell count reached approximately 10 × 10^6^ cells per ml, a portion of it was taken and diluted back to ∼0.8 × 10^6^ cells/ml. 250 ml of diluted culture was dispensed in a new bottle and 30 ml in a new TubeSpin as in step 3 above. This has been repeated 4 times in this study.
6. Growth pattern is monitored for over 600 h (25 days).

Please note maximum attainable cell density depends on cell line and media used.

**Fig. 2:**
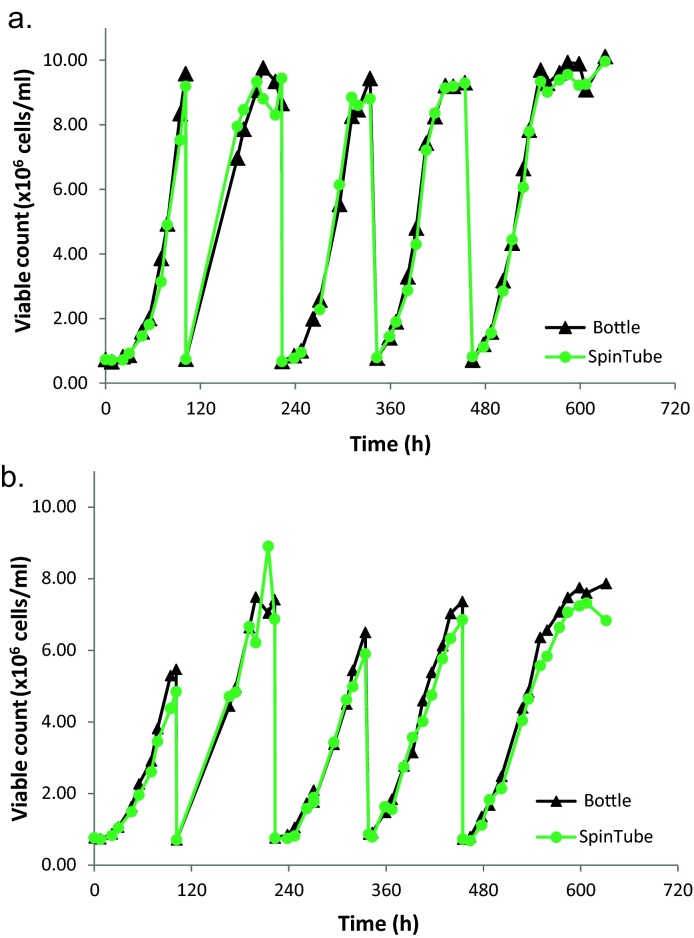
Growth of Sf9 (a) and Sf21 (b) cells in Bottle and TubeSpin.

Growth of both cell lines follow similar pattern (Fig. 2) which approves the use of TubeSpin and bottle for high fill volume insect cell culture.

## Additional information

### Background

Shaking bioreactors are widely used in academia and in bio-industry for screening and bioprocess development [1]. Very different operating conditions are used to perform such experiments introducing large discrepancy from one lab to another. The remarkable fact is “most shakers appear to run at standard speeds, often with no indication that the conditions used are optimal” [2]. This is even more prevalent in insect and mammalian cell cultures. Furthermore, the ratio of culture vessel to working culture is very high (5:1 or more) which makes it difficult to scale up without further optimization. For this reason insect cell culture is tedious and labor intensive.

We have excelled in expression of many human proteins in insect cells over past years. Small scale screening using insect cell expression system is no more a limiting factor and has been described [3]. The routine requirement for us is to screen number of targets and constructs in parallel for protein production. Small scale (∼30 ml) and Preparative scale expression (for e.g. 0.25–5 L) resulting few hundred microgram to mg of protein is more and more on demand. This allows to characterize target protein early on and to identify the right candidate to scale up. However, use of multiple vessels requires large footprints in terms of incubator space and is labor intensive. The use of reagent bottles described here helps to address it.

Use of new technique to culture insect cells raises a discussion about monitoring culture parameters online. Online monitoring of dissolved oxygen (DO) and pH in small scale is possible which has been described by Kensy et al. for *Escherichia coli* culture [4]. In case of mammalian culture, monitoring the effect of shaking and aeration in suspension has been described by Tissot et al. [5] and Zhang et al. [6]. It is practically not feasible for every lab to monitor such parameters for different scale of culture they use. Here we propose a simple method of monitoring growth parameters such as viable count, viability and diameter of insect cells grown in different vessel with different shaking speed to identify the best possible growth condition.

In this work, we have demonstrated that bottles can support insect cell growth robustly. Growth parameters of insect cells such as viability above 90%, consistent cell diameter and projected doubling time were the pre-requisites before testing these bottles for protein expression. Various targets of medical importance were expressed. Targets were purified for biochemical, biophysical and X-ray crystallography studies (see Table 2 for some examples of purified protein yields). Cell culture in bottle is suitable for all routine insect cell expression work e.g. Expression of different target protein, co-expression of target protein with inhibitors, expression of membrane protein and metabolic labeling of target protein. For most of expression studies, bottles with nominal volume of 1 L, 2 L or 5 L were used with culture volume of 0.9 L, 1.8 L and 4.9 L, respectively.

We have identified an innovative method of growing insect cells to increase throughput required for our purpose. Tubespin can be used for small volume (10–30 ml) and bottles for higher volume (250 ml to 4.9 L) culture. The yield of heterologous protein from bottle culture and conventional Erlenmeyer culture was comparable. It is noteworthy to mention here that bottles are significantly cheaper than disposable Erlenmeyer flask and can also be re-used multiple of times. With the use of bottles for 3 years we can say that culturing insect cells in Erlenmeyer or other devices with more air space and less culture volume is just a myth. This has improved the throughput and consistency with reduction in running cost. The method can be adapted in any conventional cell culture lab without any major modification of the facilities.

Useful tips:

1. While optimizing and monitoring growth behavior, besides live cell count, other parameters should also be monitored. Cell diameter, uniform distribution of cells in term of size and shape, clump or aggregation, granular appearance in cells, collectively give the status of cells. Vi-Cell counter or similar devices are very useful to observe these properties. However, in the absence of such instrument, observation under microscope serves the purpose.
2. While diluting cells from high density culture to low density culture, you might see temporary drops in viability and difference in diameter. If this happens, check cells 6–8 h or overnight after dilution to make sure that it follows normal growth. This varies with different media formulation.
3. Some medium cannot support high-density cell culture compared to others. For example cells can be maintained at density of (11–14) × 10^7^ in Sf900III medium. In other medium the limit might be around 7 × 10^6^ or less.
4. The value such as culture volume, shaking speed and the rotating diameter presented here is just a representation of our lab. This has to be optimized first for new lab set-up.
5. Shaking incubator with two different rotating diameters is not a pre-requisite for this work. It is to inform that one needs to adjust shaking speed according to rotating diameter to achieve optimum growth.
6. If aeration seems limiting for expression of some target, cells can be infected at high density and less volume. Culture can be diluted to desired density 6–8 h post infection. Oxygen demand of cells is high at the initial stage of infection.
7. Starter culture in small volume can be initiated in the same vessel and passaged by diluting until desired volume is achieved. This minimizes possible contamination risk during transfer from one vessel to another. Shaking speed needs to be adjusted for different volume to avoid foam formation.

**Table:** 

Fill volume	Shaking speed with orbital diameter of	
	25 mm	50 mm
Less than half	90 rpm	90 rpm
About half	100–110 rpm	100–110 rpm
More than half	≥ 170 rpm	120 rpm

*Note*: Above values are presented as guidelines. Therefore, it is recommended to optimize shaking speed in your lab set up.

## Figures and Tables

**Table 1:** Growth of Sf21 insect cells in different culture vessel.

Expt. No.	Nominal vessel volume (V1)	Culture volume (V2)	Ratio (V1:V2)	Shaking speed (rpm)	Shaking diameter
a.	250 ml Erlenmeyer^a^	50 ml	5:1	90	50 mm
b.	250 ml Erlenmeyer	250 ml	1:1	230	25 mm
c.	250 ml bottle	250 ml	1:1	230	25 mm
d.	50 ml TubeSpin	30 ml	1.7:1	230	25 mm

Shaking speed chosen for Expt. b, c and d was the optimum speed identified for incubator with 25 mm rotating diameter. Shaking speed of 170 and 230 were good to keep cells in suspension, however, the later gave better cell viability.

a Standard condition for the growth of insect cells used in most of the lab.

**Table 2:** Yield of different protein in Erlenmeyer flasks and bottle.

Target	Yield in Erlenmeyer (mg/L)^a^	Yield in bottle (mg/L)^b^	Cell line
Kinase domain (kinase)	30.0	32.0	Sf9
	20.0	15.0	Sf21
Kinase-1	0.8	1.5	Sf9
Kinase-2	0.5	0.5	Sf9
Kinase-3	8.8	8.3	Sf21
Kinase-4	10.8	9.1	Sf21
Kinase-5	1.5	1.7	Sf21
Receptor	8.4	10.0	Sf9
Receptor-subunit (secreted)	0.3	0.3	Sf9

a Ratio of nominal vessel volume:culture volume = 5:1.

b Ratio of nominal vessel volume:culture volume = 1:1.
